# Metabolomic and transcriptomic analyses jointly reveal the mechanism underlying the reddening of *Chimonanthus praecox* stamens

**DOI:** 10.3389/fpls.2024.1491246

**Published:** 2024-11-20

**Authors:** Bin Liu, Huafeng Wu, Yinzhu Cao, Guanpeng Ma, Xiaowen Zheng, Haoxiang Zhu, Xingrong Song, Shunzhao Sui

**Affiliations:** ^1^ Chongqing Engineering Research Center for Floriculture, Key Laboratory of Agricultural Biosafety and Green Production of Upper Yangtze River (Ministry of Education), College of Horticulture and Landscape Architecture, Southwest University, Chongqing, China; ^2^ Institute of Horticulture, Guizhou Academy of Agricultural Sciences, Guiyang, Guizhou, China; ^3^ College of Horticulture and Landscape Architecture, Southwest University, Chongqing, China; ^4^ Garden and Flower Research Center, Horticultural Research Institute of Sichuan Academy of Agricultural Science, Chengdu, Sichuan, China

**Keywords:** *Chimonanthus praecox*, stamen, anthocyanin biosynthesis, UPLC-MS/MS, transcriptome sequencing

## Abstract

**Introduction:**

Flower characteristics are crucial ornamental and reproductive traits in *Chimonanthus praecox*. Over its long cultivation history, variations have been observed in the floral organs, primarily in the petals, with limited reports on stamen traits. Stamen variation, integral to the mating system, can enhance the plant’s ornamental value and directly impact its reproductive success.

**Methods:**

This study is the first to report the phenomenon of red coloration in *C. praecox* stamens. Using UPLC-MS/MS, we analyzed the types and quantities of major metabolites in stamens of different colors.

**Results:**

Our results indicated that the red coloration was primarily due to the accumulation 42 on of high levels of anthocyanins, specifically cyanidin 3-O-rutinoside and cyanidin 3-O-glucoside. Transcriptomic sequencing identified 63 differentially expressed genes (DEGs) related to the anthocyanin biosynthetic pathway, most showing peak expression during the bud stage. The results of the metabolite analysis and transcriptomic sequencing were similar to those of previous studies on petal reddening, suggesting a close relationship between the mechanisms of stamen and petal reddening.

**Discussion:**

This study elucidated the mechanism of stamen reddening in *C. praecox*, expanding the species’ genetic resources and offering insights into color changes across floral tissue..

## Introduction

*Chimonanthus praecox*, a prized ornamental unique to China, is a rare and exceptional winter-flowering tree with a long cultivation history (Sui et al., 2015; Shang et al., 2020). As a landscape plant, *C. praecox* can be admired for up to 5 months in gardens, and its cut flowers can last over 3 weeks in a vase. When in bloom, the tree is covered with golden-yellow flowers with a rich, fragrant aroma (Chen, 2012; Sui et al., 2012). The floral traits of *C. praecox*, key to its ornamental appeal, have varied significantly over centuries of cultivation (Zhu et al., 2024). These traits, which are reproductive in nature, show notable variability both between populations and within a single population, serving as an adaptation to environmental changes (Carr and Fenster, 1994; Williams and Conner, 2001; E-Vojtkó et al., 2022). *C. praecox* is a predominantly outcrossing species with both dioecious and heterostylous traits that rely on insect pollination (Zhou et al., 2006). Outcrossing not only maintains high genetic diversity within the plant population but also enhances the adaptability of plants to environmental conditions. It is considered the most advantageous mating system for flowering plants because it helps to avoid inbreeding depression (Bellusci et al., 2009; Travers et al., 2018). It is widely believed that most plant reproductive traits, particularly floral structural traits, are the result of natural selection mediated by pollinators (Carr and Fenster, 1994; Williams and Conner, 2001).

The primary morphological traits of plant flowers have evolved under selective pressures aimed at enhancing male function, specifically the dissemination of pollen to receptive stigmas (Barrett, 2002, 2003). As direct participants in male function, stamens exhibit certain variations in morphology and structure under these selective pressures. Pollen grains from stamens often serve as attractants for pollinators, such as when pollen is offered as a reward (Ashman, 2000). Approximately 20,000 flowering plant species exclusively use pollen as an attractant for insects (Lu et al., 2009). Additionally, anthers may use color as an indicator to attract pollinators (Lau and Galloway, 2004). Consequently, differences in anther morphology and color can affect the ability of flowers to attract pollinators and influence pollinator behavior (Escaravage et al., 2001; Kudo, 2003). Anther color differentiation has been widely observed in various plant families, including Acanthaceae, Balsaminaceae, Commelinaceae, Passifloraceae, Fabaceae, and Lythraceae. This differentiation primarily involves the presence of brightly colored stamens or anthers to attract pollinators, thereby enhancing pollen output (Ren, 2009).

The color variation of *C. praecox* flowers is mainly expressed in the tepals, and the study of *C. praecox* flower color is also mainly focused on the tepals (Zhou et al., 2007; Ge et al., 2008; Shen et al., 2021). The outer petals of *C. praecox* have evolved into scales, with the middle petals typically yellow or light yellow and occasionally yellowish-white or yellow-green. The inner petals, located inside the flower, display a wide range of colors, including purple, red, and yellow. Yellow petals primarily derive their color from flavonols, while red petals contain anthocyanins, with the intensity of red coloration directly linked to anthocyanin content (Shen et al., 2022). Anthocyanins are water-soluble flavonoid compounds widely found in plant roots, stems, leaves, flowers, fruits, and seeds, and contribute to red, blue, and purple hues (Wu et al., 2023). Targeted flavonoid metabolomic analysis of different flower color types in *C. praecox* has shown that the characteristic red metabolite in red-flowered varieties is chrysanthemin, including chrysanthemin-3-O-glucoside, chrysanthemin-3-O-rutinoside, and chrysanthemin-3-O-galactoside (Shen et al., 2023). Color variation in *C. praecox* (‘Meiren Zui’) results from the combined effects of anthocyanins, flavonoids, and chlorophyll, with anthocyanin content being the primary determinant (Xie et al., 2023).

The physiological mechanisms affecting the color change of *C. praecox* petals, including pigment types and concentrations, as well as the molecular mechanisms involving major metabolic pathways and key gene functions, have been well-studied (Zhou et al., 2007; Ge et al., 2008; Shen et al., 2021). However, the reddening of *C. praecox* stamens and its underlying mechanisms remain unexplored. It is unclear whether the processes responsible for stamen reddening are similar to those for petal reddening. The flower characteristics of *C.praecox*, as important ornamental and reproductive traits, make the discovery of red-stamened germplasm a key resource for modern plant breeding (Zhu et al., 2022; Zheng et al., 2024). Additionally, the reddening of the stamens may affect pollinator behavior and pollination rates, which has significant biological implications (Ren, 2009). In this study, we report for the first time the phenomenon of reddening in *C. praecox* stamens. Using UPLC-MS/MS and transcriptomic sequencing technologies, we conducted preliminary analyses to identify the main substances and molecular mechanisms responsible for stamen reddening, thereby laying a foundation for further research on the regulatory mechanisms of stamen color changes in *C. praecox*.

## Materials and methods

### Plant materials

This study investigated two *C. praecox* plant varieties: one with red stamens (CP-PR) and the other with yellow stamens (CP-N), both cultivated in the germplasm nursery of the College of Horticulture and Landscape Architecture at Southwest University. Detailed names and geographical information are provided in **Supplementary Table S1**. In December 2023, stamens at different developmental stages of the corresponding varieties were collected. Petals and other floral parts were removed, leaving only stamens. Twenty samples were collected and stored in centrifuge tubes, with technical replicates performed in triplicate. Samples were immediately treated with liquid nitrogen and stored in a –80°C ultra-low temperature freezer at the Flower Laboratory of Southwest University.

### Determination of anthocyanin components

The collected CP-PR and CP-N samples were vacuum-freeze-dried and ground into powder using a ball mill (30 Hz, 1.5 min). Fifty milligrams of the powdered sample were dissolved in 500 μL of extraction solvent (50% methanol aqueous solution containing 0.1% hydrochloric acid). The mixture was vortexed for 5 min, ultrasonicated for 5 min, and then centrifuged for 3 min (12,000 r/min, 4°C). The supernatant was collected, the extraction was repeated once, and the two supernatants were combined. The sample was filtered through a micropore membrane (0.22 μm pore size) and stored in an injection vial for UPLC-MS/MS analysis. The data acquisition system consisted primarily of an Ultra Performance Liquid Chromatography (UPLC) system (ExionLC™ AD) and a Tandem Mass Spectrometry (MS/MS) system (QTRAP^®^ 6500+).

### Transcriptome sequencing, assembly, annotation, and analysis

Total RNA was extracted using the RNAprep Pure Kit (TianGen, Beijing, China) according to the manufacturer’s instructions. RNA quality was assessed using 1% agarose gel electrophoresis and a NanoDrop ND-1000 spectrophotometer (Thermo Fisher Scientific, Wilmington, MA, USA). The library was constructed by enriching mRNA with oligo (dT) magnetic beads, then synthesizing first-strand cDNA with reverse transcriptase. This cDNA was used to create double-stranded cDNA, followed by end repair and addition of a single ‘A’ nucleotide at the 3’ end. An aptamer was attached to the cDNA, and the library’s quality was assessed by amplifying it with phi29 DNA polymerase. DNA nanoballs (DNBs) containing the library were sequenced on the BGIseq500 platform (BGIseq500, Shenzhen, China), generating 100 base reads. Data were processed in triplicate, with raw data filtered using SOAPnuke (v1.5.2) (Chen et al., 2018) to exclude aptamer-containing reads, reads with >10% unknown bases, and reads with >50% low-quality bases. Clean reads were saved in FASTA format. Annotations were performed using eight major functional databases (KEGG, GO, NR, NT, KOG, TF, Swiss-Prot, and Pfam). Differential gene expression analysis within groups was conducted using DESeq (Wang et al., 2010), with fold change ≥ 2 and adjusted p-value ≤ 0.001. Cluster analysis of genes with similar expression profiles was performed using Mfuzz software (v2.6.0) (Kumar and Futschik, 2007). Graphical representations were generated using the online tool SRplot (Tang et al., 2023).

### Quantitative real-time PCR

cDNA was synthesized using an all-in-one first-strand synthesis master mix (with dsDNase) (Jiangsu Yugo Biotechnology Co., Ltd.). Quantitative PCR (qPCR) was performed using the 2×TSINGKE^®^ Master qPCR Mix (SYBR Green I) kit (Beijing Tsingke Biotechnology Co., Ltd.), following the manufacturer’s instructions. This procedure included three biological replicates and three technical replicates. Primer sequences are listed in **Supplementary Table S2**. Reference genes were selected based on the literature (Chen et al., 2022). Relative expression levels of target genes were calculated using the 2^-ΔΔ^CT method (Livak and Schmittgen, 2001).

## Results

### Morphological observation

**Figure 1:**
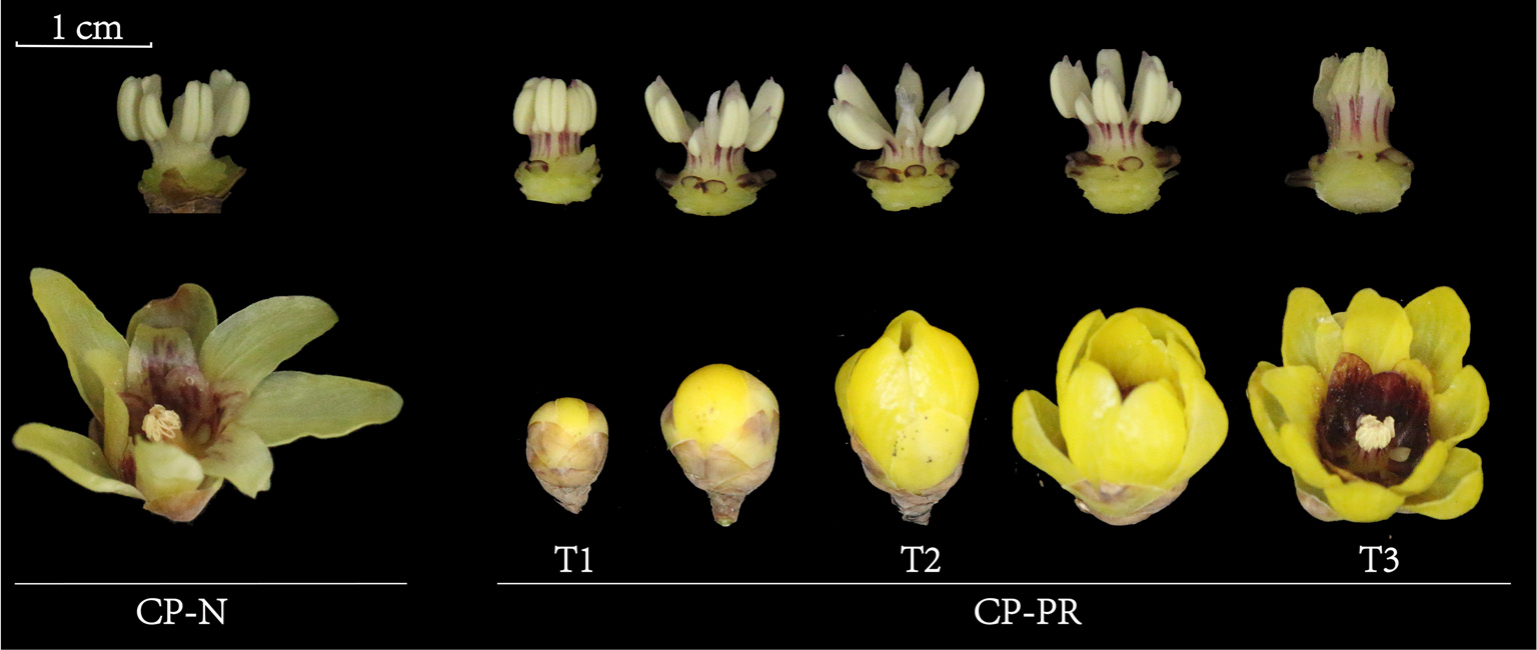
Morphological observations of *Chimonanthus praecox* varieties with red stamens (CP-PR) and varieties with yellow stamens (CP-N). CP-PRT1 represents the bud stage, CP-PRT2 represents the early bloom stage, CP-PRT3 represents the full bloom stage, and CP-N represents the full bloom stage.

Morphological observations were conducted on the stamens of CP-PR and CP-N varieties, as shown in **Figure 1**. In the CP-N group, the entire stamens appeared uniformly light yellow. Conversely, in the CP-PR variety, the filaments of the stamens showed a distinct red color, and the tips of the anthers showed a light red color. The redness at the tips of the anthers faded slightly as the flowers opened fully. The filaments extended slightly during flowering, and the outer parts of the filaments maintained a noticeable striped red coloration throughout the flowering period.

### Quantitative analysis of anthocyanins based on UPLC-MS/MS

**Figure 2:**
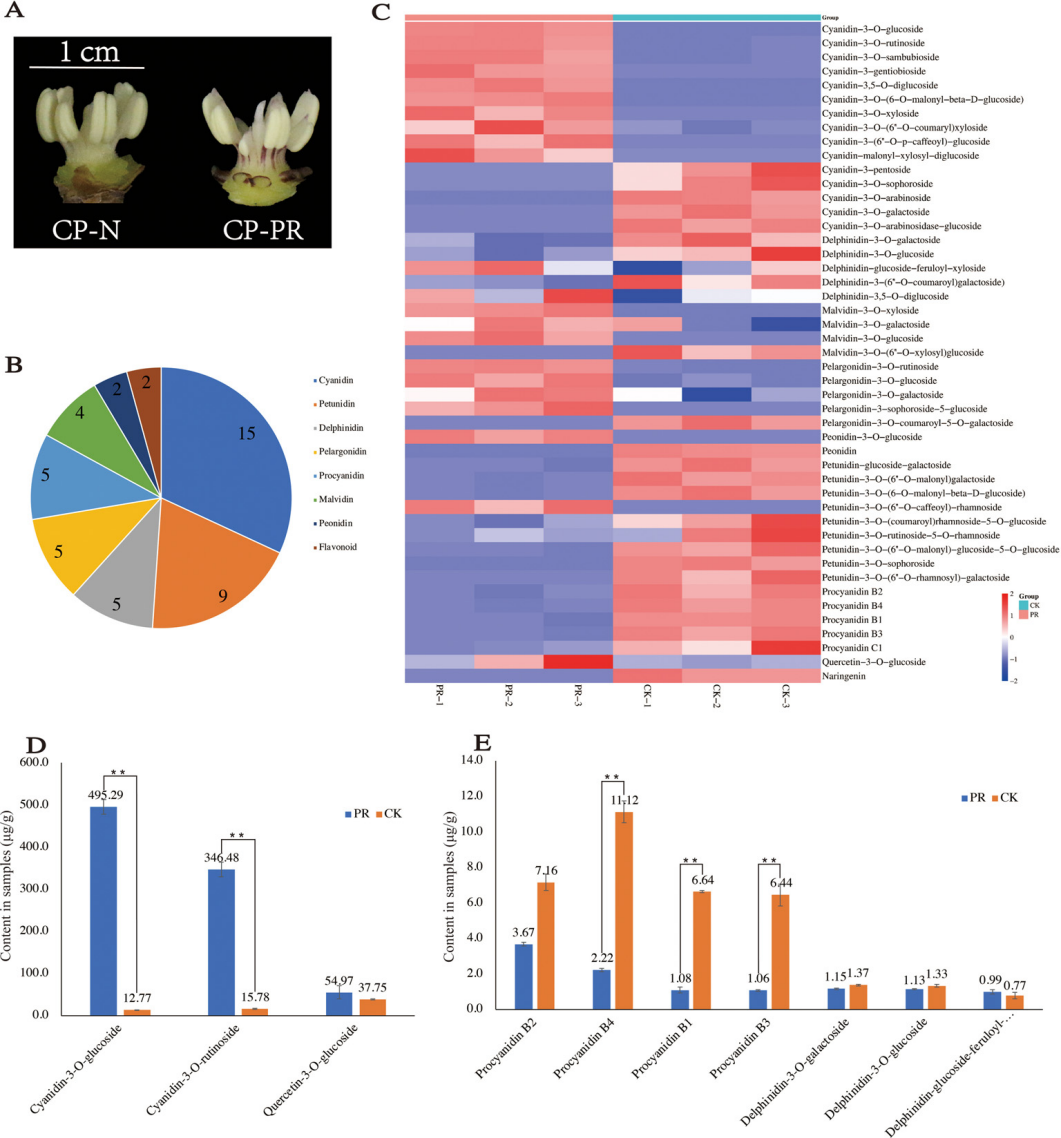
Quantitative analysis results of anthocyanins. **(A)** CP-PR and CP-N varieties used for anthocyanin quantification. **(B)** Classification of anthocyanin substances detected. **(C)** Differential analysis of anthocyanin content between CP-PR and CP-N. **(D)** Significant differences in the content of specific substances between CP-PR and CP-N. **(E)** Significant differences in the content of additional substances between CP-PR and CP-N. ** indicates a significant difference between the two at the p < 0.05 level.

Quantitative detection of anthocyanins in the stamens of CP-PR and CP-N at the early bloom stage was performed to analyze differences in anthocyanin content (**Figure 2A**). Eight major types of anthocyanins were identified (**Supplementary Table S3**). The most prevalent was cyanidin (15), followed by petunidin (9). Delphinidin, pelargonidin, and procyanidin were detected in five forms, whereas malvidin was detected in four forms. Peonidins and flavonoids were each detected in two forms each (**Figure 2B**). Analysis of the differences in the content of various substances in CP-PR and CP-N showed that cyanidin (10/15), pelargonidin (4/5), and malvidin (3/4) had higher concentrations in CP-PR, whereas petunidin (8/9), delphinidin (3/5), and procyanidin (5/5) had higher concentrations in CP-N (**Figure 2C**). The 49 compounds detected were observed for cyanidin-3-O-rutinoside (cyanidin), cyanidin-3-O-glucoside (cyanidin), and quercetin-3-O-glucoside (flavonoid). Specifically, cyanidin-3-O-rutinoside and cyanidin-3-O-glucoside were significantly higher in CP-PR, with concentrations of 346.48 μg/g and 495.29 μg/g, respectively, compared to CP-N. The content of quercetin-3-O-glucoside content was not significantly different between the CP-PR and CP-N groups (**Figure 2D**). Procyanidin B1/B2/B3/B4 and delphinidin-3-O-galactoside/delphinidin-3-O-glucoside were found in higher concentrations in the CP-N group. Notably, procyanidin B1/B3/B4 levels differed significantly between CP-PR and CP-N (**Figure 2E**).

### Basic data analysis, annotation information, and expression clustering

**Figure 3:**
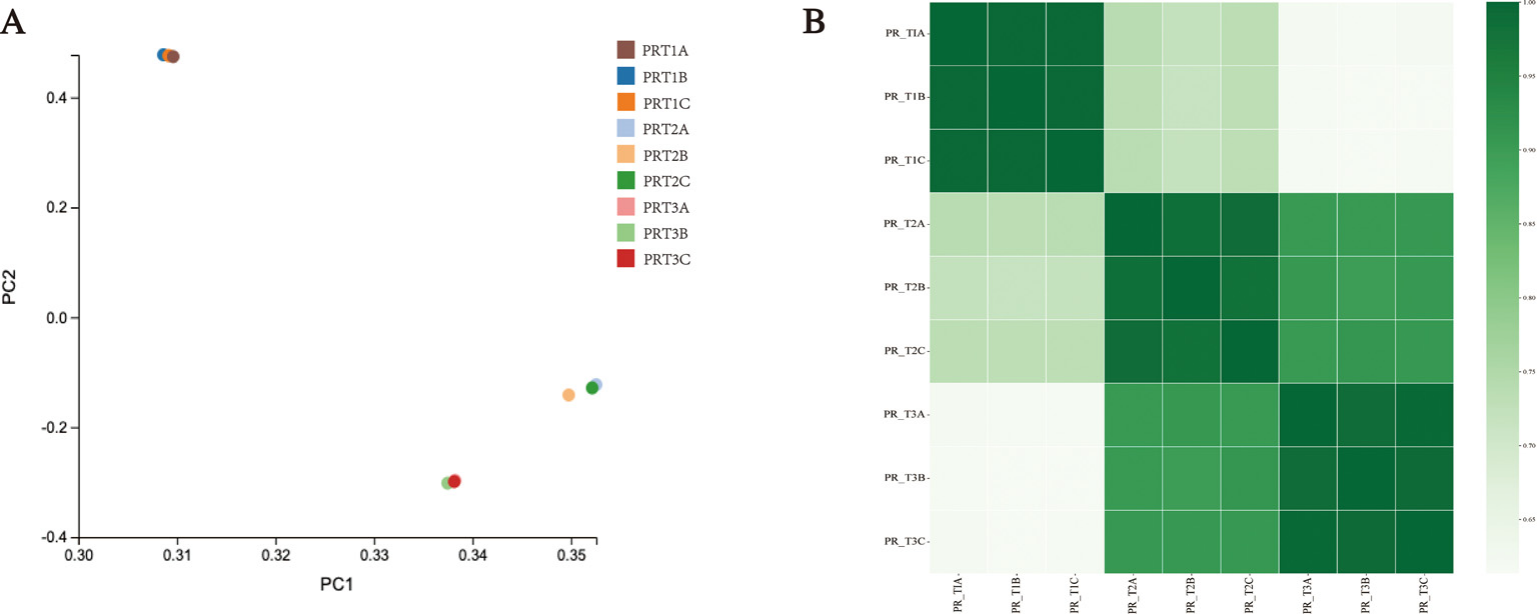
Overview of transcriptome data. **(A)** Principal component analysis (PCA) results. **(B)** Sample correlation analysis results.

In order to investigate the intrinsic mechanism of stamen red color formation, transcriptome sequencing was performed on the stamens of *C. praecox* at the bud (CP-PRT1), early bloom (CP-PRT2), and full bloom (CP-PRT3) stages. Nine samples were analyzed, with an average data output of 6.62 Gb per sample (**Supplementary Table S4**). In total, 30,007 expressed genes were detected, including 22,152 known genes and 7,855 newly predicted genes, corresponding to 14,343 new transcripts. The known genes had a total of 25,509 transcript lengths, with 78.80% of transcripts longer than 500 bp. Among these, 6,641 transcripts ranged from 500–1,000 bp, 9,262 transcripts ranged from 1,000–2,000 bp, and 4,197 transcripts were longer than 2,000 bp (**Supplementary Table S5**). Principal component analysis (PCA) indicated that samples within the same group were closely related (**Figure 3A**), and correlation analysis among the samples showed good correlation (**Figure 3B**). Annotation results using eight major public databases showed that the NR database annotated the highest number of genes (30,387), followed by the GO database (26,418 genes). The numbers of annotated genes in the KEGG, NT, KOG, TF, Swiss-Prot, and Pfam databases were 10,109, 22,036, 18,014, 14,232, 22,430, and 8,177, respectively (**Supplementary Figure S1A**). Venn diagram analysis of annotations from the NR, GO, KEGG, NT, KOG, TF, and Pfam databases revealed that 893 genes could be annotated using multiple databases (**Supplementary Figure S1B**).

### Differentially expressed gene analysis

**Figure 4:**
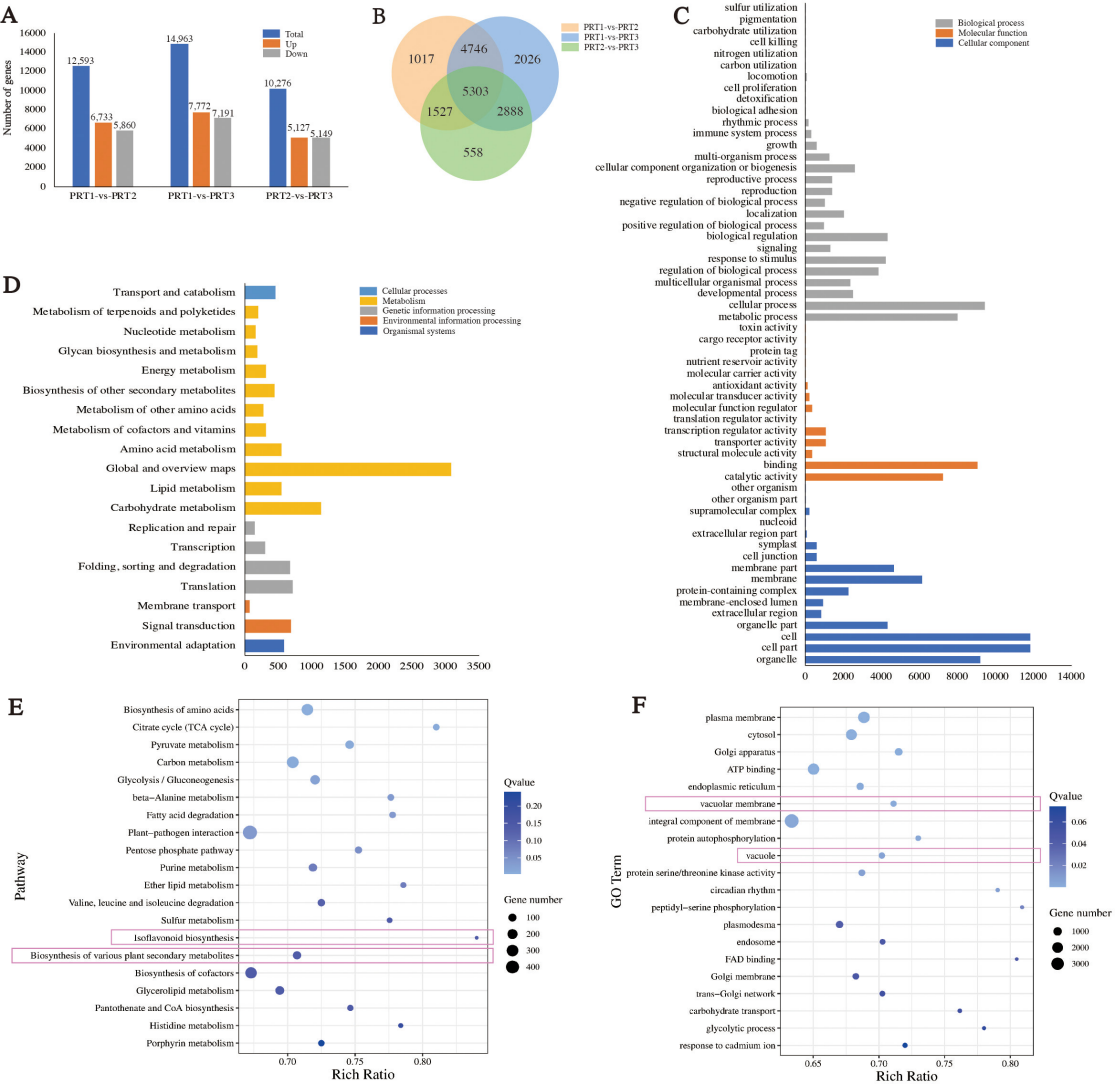
Analysis of differentially expressed gene (DEGs). **(A)** Analysis of upregulated and downregulated DEGs between groups. **(B)** Venn diagram of DEGs between groups. **(C)** GO classification results of DEGs. **(D)** KEGG classification results of DEGs. **(E)** KEGG enrichment results for DEGs. **(F)** GO enrichment results for DEGs.

Differential expression analysis was conducted on the genes detected in CP-PRT1, CP-PRT2, and CP-PRT3 samples. In the comparison between CP-PRT1 and CP-PRT2, 12,593 differentially expressed genes (DEGs) were identified, with 6,733 upregulated and 5,860 downregulated genes. In the comparison between CP-PRT1 and CP-PRT3, 14,963 DEGs were identified, with 7,772 upregulated and 7,191 downregulated genes. In the comparison between CP-PRT2 and CP-PRT3, 10,276 DEGs were identified, with 5,127 upregulated and 5,149 downregulated genes (**Figure 4A**). A total of 18,605 DEGs were detected across CP-PRT1, CP-PRT2, and CP-PRT3, of which 5,303 genes were differentially expressed in all three groups. Additionally, 1,017, 2,026, and 558 DEGs were unique to the CP-PRT1 vs. CP-PRT2, CP-PRT1 vs. CP-PRT3, and CP-PRT2 vs. CP-PRT3 comparisons, respectively (**Figure 4B**). KEGG and GO classification and enrichment analyses were performed on 18,605 DEGs. GO classification revealed that these genes could be categorized into three major categories: biological processes (28), molecular functions (14), and cellular components (16) (**Figure 4C**). The KEGG classification showed that these genes could be divided into five major categories: cellular processes (1), metabolism (11), genetic information processing (4), environmental information processing (2), and organismal systems (1) (**Figure 4D**). Among the top 20 enriched KEGG pathways, those related to anthocyanin metabolism, such as isoflavonoid biosynthesis and biosynthesis of various plant secondary metabolites, were identified (**Figure 4E**). Following anthocyanin synthesis, the pigment is transported to the vacuole, contributing to coloration. In the top 20 GO enrichment pathways, vacuolar membrane and vacuoles were associated with anthocyanin pigmentation (**Figure 4F**). Time-series clustering analysis of the 18,605 DEGs identified nine clusters. For example, Cluster 1 genes showed an upregulation trend during the flowering period, whereas cluster 2 genes exhibited a downregulation trend with changes in flowering time (**Supplementary Figure S2**).

### Analysis of anthocyanin biosynthesis pathways

**Figure 5:**
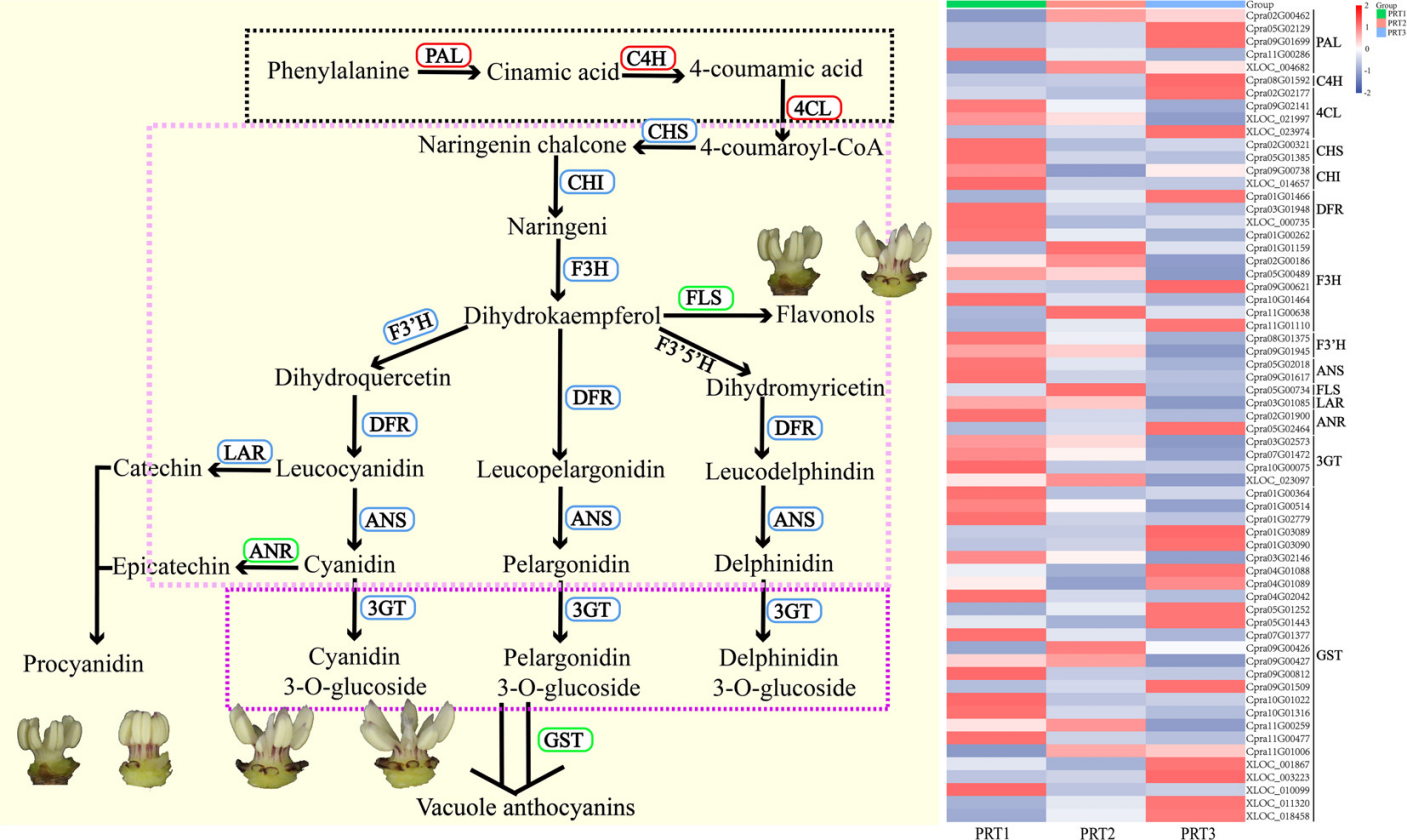
Analysis of DEGs in anthocyanin metabolism pathways. PAL (5): phenylalanine ammonia-lyase; C4H (1): cinnamic acid 4-hydroxylase; 4CL (4): 4-coumaric acid-CoA ligase; CHS (2): chalcone synthase; CHI (2): chalcone isomerase; F3H (8): flavanone 3-hydroxylase; F3’H (2): flavonoid 3’-hydroxylase; F3’5’H: flavonoid 3’,5’-hydroxylase; DFR (3): dihydroflavonol 4-reductase; ANS (2): anthocyanidin synthase; 3GT (4): anthocyanidin 3-O-glucosyltransferase; GST (26): glutathione S-transferase.

Among the 18,605 DEGs, 63 were related to anthocyanin metabolism, categorized into four major groups and 14 subcategories of enzyme genes (**Supplementary Table S6**). These include: Phenylpropanoid biosynthesis (ko00940) with *PAL* (5), *C4H* (1), and *4CL* (4); Flavonoid biosynthesis (ko00941) with *CHS* (2), *CHI* (2), *DFR* (3), *F3H* (8), *F3’H* (2), *FLS* (1), *ANS* (2), *LAR* (1), and *ANR* (2); Anthocyanin biosynthesis (ko00942) with *3GT* (4); and transporter-related genes for *glutathione S-transferase* (*GST*) (26) (**Figure 5**). Differential expression analysis revealed that three genes in the phenylpropanoid biosynthesis pathway (ko00940) showed a predominant upregulation trend with changes in flowering time, whereas eight genes in the flavonoid biosynthesis pathway (ko00941) and anthocyanin biosynthesis pathway (ko00942) generally exhibited a downward trend. Notably, *FLS* (1) showed the highest expression in the middle CP-PRT2 stage. Additionally, the number of upregulated and downregulated genes for *ANR* (1) and transporter-related *GST* (26) was approximately equal to the change in flowering time (**Figure 5**). No expression of the *F3’5’H* gene was detected in the DEG screening process for anthocyanin metabolism. In the CP-PR samples, the average content of delphinidin-3-O-glucoside was 1.15 μg/g, while the highest average content of cyanidin-3-O-glucoside was 495.29 μg/g (**Figures 2**, **5**). To validate the transcriptome data, five DEGs (*Cpra02G00321/CHS*, *Cpra01G00262/F3H*, *Cpra08G01375/F3’H*, *Cpra05G02018/CpANS1*, *Cpra05G02018/CpANS2*) from the anthocyanin biosynthesis pathway were confirmed using real-time quantitative PCR. The results of the real-time quantitative PCR were consistent with the transcriptome data (**Supplementary Figure S3**), indicating the reliability of the transcriptome data.

## Discussion

*C. praecox*, commonly known as wintersweet, is a rare and valuable winter-flowering tree of significant ornamental, medicinal, and cultural importance. Through cultivation, numerous outstanding cultivars have been developed (Chen, 2012; Zhu et al., 2024). Zhao (2008) surveyed *C. praecox* germplasm resources across 13 provinces in China and found that these resources were primarily distributed south of the Qinling Mountains and east of the Hengduan Mountains, with a broad cultivation area (Zhao, 2008). Subsequent studies investigated and classified *C. praecox* varieties from various regions, including Sichuan, Hangzhou in Zhejiang, Yanling in Henan, and Wuhan in Hubei (Wu et al., 2009; Lu et al., 2010; Lu and Ren, 2011; Lu and Rong, 2012; Lu and Xie, 2012; Song et al., 2012). Flower color plays a crucial role in the ornamental and economic value of many plants, and breeding new flowers is one of the main goals of ornamental plant breeding. The formation of flower color has also been the focus of research for centuries (To and Wang, 2006; Sawada et al., 2019; Mekapogu et al., 2020). *C. praecox* predominantly exhibits yellow flowers, with the inner tepals containing primarily cyanidin glycosides (cy-type pigments), resulting in varying degrees of red coloration (Zhou and Chen, 2010; Shen et al., 2022). The flower organs of *C. praecox* are crucial to cultivar classification, with varieties often distinguished by the color of the inner tepals. They are categorized into the Concolor, Intermedius, and Patens groups. The recent development of the red-flowered Hongyun (*C. praecox* ‘Hongyun’) cultivar, classified in the Rubrum Group, has further enriched the diversity of *C. praecox* (Shen et al., 2021).

Flower color is one of the most important phenotypic traits of ornamental plants and serves as a key indicator of their aesthetic and commercial value. Flowers are crucial reproductive organs in plants, and flower color plays a vital role in attracting pollinators and facilitating sexual reproduction (Li and Yue, 2023). Currently, color variations in the floral organs of *C.praecox* have only been observed in the petals. This study is the first to report changes in the color of the stamens, which not only enriches the diversity of *C.praecox* varieties but also provides a research foundation for modern plant breeding of *C.praecox*, enabling precise improvements through gene editing and other technologies (Tan et al., 2023; Zhang et al., 2024). This may have significant implications for studying the reliance of *C.praecox* on insect pollination for cross-breeding (Ren, 2009). Through morphological observation, we found that the stamens exhibited a red color similar to that of the tepals. Red coloration in the tepals is primarily due to the differential accumulation of anthocyanins. Anthocyanins possess strong antioxidant properties, which can enhance visual quality, reduce photo-inhibition under intense light, minimize damage from ultraviolet radiation, and improve the plant’s resistance to pests and diseases (Dixon et al., 2002; Zhou, 2021). Over 550 types of anthocyanins have been identified, with most derived from six common anthocyanins: cyanidin, delphinidin, malvidin, peonidin, pelargonidin, and petunidin (You et al., 2011). In this study, the analysis of anthocyanin content in *C. praecox* stamens revealed eight major categories of 49 substances. The red stamens primarily accumulate cyanidin-related compounds, whereas the yellow stamens mainly accumulate flavonoids. Research by Ge et al. (2008) and Li and Yue (2023) on *C. praecox* flower color and pigments identified two anthocyanins and three flavonols in the red inner tepals: cyanidin 3-O-glucoside and cyanidin 3-O-rutinoside, and quercetin 3-O-rutinoside, kaempferol 3-O-rutinoside, and quercetin aglycone (Ge et al., 2008; Yu, 2013). Zhou et al. (2007) study on *C. praecox* flower color found that the pigment content in the inner tepals correlated positively with color intensity (Zhou et al., 2007). Shen identified cyanidin derivatives as key metabolic compounds responsible for the red coloration of *C. praecox* tepals, including cyanidin 3-O-glucoside, cyanidin 3-O-rutinoside, and cyanidin 3-O-galactoside (Shen et al., 2021). In the present study, the pigment analysis showed similar results, with higher levels of cyanidin 3-O-rutinoside, cyanidin 3-O-glucoside, and other substances in red stamens than in yellow stamens. In yellow stamens, the highest content was found for quercetin-3-O-glucoside, with no significant difference compared with red stamens. Anthocyanins were also detected in the yellow stamens. These findings suggest that the accumulation of cyanidin 3-O-rutinoside and cyanidin 3-O-glucoside is the main metabolic driver of red coloration in *C. praecox* stamens, and that flavonoid synthesis is not affected by anthocyanin accumulation.

The anthocyanin biosynthetic pathway is divided into four stages. The first stage is the phenylpropanoid biosynthesis pathway (ko00940), which primarily provides precursor substances for the downstream flavonoid pathway. In this stage, phenylalanine is converted to cinnamoyl-CoA through the actions of *phenylalanine ammonia-lyase* (*PAL*), *cinnamate-4-hydroxylase* (*C4H*), and *4-coumarate-CoA ligase* (*4CL*). The second stage is the flavonoid biosynthesis pathway (ko00941), where colorless flavonoid precursors are converted into colored anthocyanins. This transformation occurs through the actions of *chalcone synthase* (*CHS*), *chalcone isomerase* (*CHI*), *flavanone-3-hydroxylase* (*F3H*), *flavanone 3’-hydroxylase* (*F3’H*), *flavanone 3’,5’-hydroxylase* (*F3’5’H*), *dihydroflavonol 4-reductase* (*DFR*), and *anthocyanidin synthase* (*ANS*), resulting in the production of cyanidin, pelargonidin, and delphinidin. Additionally, leukocyanidin is converted into catechin and epicatechin by *LAR*, and cyanidin is converted into procyanidin by *ANR*. The third stage involves the anthocyanin biosynthesis pathway (ko00942). At this stage, cyanidin, pelargonidin, and delphinidin are glycosylated to form cyanidin-3-O-glucoside, pelargonidin-3-O-glucoside, and delphinidin-3-O-glucoside via anthocyanidin *3-O-glucosyltransferase* (3GT). These compounds undergo further modifications such as acylation and methylation to produce various anthocyanin glycosides. In the fourth stage, anthocyanin glycosides are transported to the vacuole via GST, where they are stored and contribute to coloration. The type of anthocyanin determines the color variation (Fan et al., 2023; Hu et al., 2023; Kang et al., 2023; Zhang et al., 2023; Ma et al., 2024). Differential gene expression analysis in the red stamens of *C. praecox* at various developmental stages showed an increasing trend in the expression of three genes (*PAL*, *C4H*, *4CL*) in the first stage. However, the expression of seven genes in the second stage (*CHS*, *CHI*, *F3H*, *F3’H*, *DFR*, *ANS*, *LAR*) and the 3GT gene in the third stage decreased. Yu found that the expression of *CpF3’H* was highest in the flower buds of red-flowered *C. praecox* (Yu, 2017). Our results are consistent with these findings, showing that this gene has the highest expression in PRT1. Yang found that *ANS* expression plays a crucial role in anthocyanin biosynthesis. *CpANS1* showed the highest expression during flower bud maturation, followed by the full bloom stage, and the lowest expression in the immature stage. Additionally, there was no competitive expression between *flavonol synthase* (*FLS*) and *anthocyanin synthase* (*ANS*) (Yang et al., 2018). Shen concluded that the red coloration of tepals was primarily due to the high expression of the structural gene *CpANS1* in the anthocyanin biosynthesis pathway, confirming that *CpANS1* is positively correlated with anthocyanin glycosides and is a key structural gene in the red coloration of *C. praecox* tepals (Shen et al., 2021).

In the present study, both ANS genes were detected during all three stages of red stamen development, with expression trends similar to those reported previously. Expression was the highest during the flower bud maturation stage (PRT1) and the lowest during the full bloom stage (PRT3). Based on these results, it can be inferred that the mechanism of red coloration in *C. praecox* stamens is similar to that in tepals, primarily due to differential gene expression in the flavonoid pathway, leading to varying anthocyanin accumulation and resulting in red coloration in the stamens. Additionally, the downregulation of genes involved in flavonoid and anthocyanin biosynthesis pathways during the development of red stamens suggests that anthocyanin accumulation is concentrated around the flower bud maturation period, with a gradual decrease in anthocyanin content from the bud stage to full bloom. The phenylpropanoid pathway is a crucial secondary metabolic pathway in plants that serves as the primary source. This pathway also participates in the synthesis of various secondary metabolites and plant hormones such as phenolic acids, flavonoids, lignin, and salicylic acid through multiple downstream branches. These compounds play a significant role in plant stress responses, growth and development, cellulose synthesis, and the accumulation of medicinal components (Dixon et al., 1996; Anwar et al., 2021). It is estimated that over 20% of secondary metabolites in cells are synthesized via this pathway (Grace and Logan, 2000). In the red stamens of *C. praecox*, the expression levels of genes in the phenylpropanoid biosynthesis pathway increased from the bud stage to full bloom, which may indicate a preparatory step for the synthesis of precursors of anthocyanins and other substances.

## Conclusion

This study is the first to report stamen reddening in *C. praecox*, enriching the genetic resources of this species. Using UPLC-MS/MS, we identified the primary metabolites in red stamens as cyanidin derivatives, specifically cyanidin 3-O-rutinoside and cyanidin 3-O-glucoside, whereas the main metabolite in yellow stamens was quercetin-3-O-glucoside, a flavonol. This significant difference in the cyanidin content is likely the main reason for the reddening of *C. praecox* stamens. Transcriptome sequencing analysis of red stamens during development revealed the differential expression of genes related to the anthocyanin biosynthesis pathway. It has been preliminarily suggested that the mechanism of red coloration in stamens is similar to that in tepals, primarily due to the differential expression of flavonoid pathway genes, leading to varied anthocyanin accumulation. These findings provide a basis for further research on the mechanisms underlying red coloration in *C. praecox* floral organs.

## Data Availability

The datasets presented in this study can be found in online repositories. The names of the repository/repositories and accession number(s) can be found below: https://www.ncbi.nlm.nih.gov/sra/PRJNA1154814, National Center for Biotechnology Information, No. PRJNA1154814.

## Supplementary material

Supplementary Figure 1 Annotation information of the transcriptome data.

Supplementary Figure 2 Time-series clustering analysis results of DEGs.

Supplementary Figure 3 Comparison of transcriptome sequencing results with real-time quantitative PCR results.
