# Supplementary figures and images for: Metabolomic and transcriptomic analyses jointly reveal the mechanism underlying the reddening of Chimonanthus praecox stamens
Source: Front Plant Sci. 2024 Nov 20;15:1491246. doi: 10.3389/fpls.2024.1491246 (PMC11618622; doi:10.3389/fpls.2024.1491246)

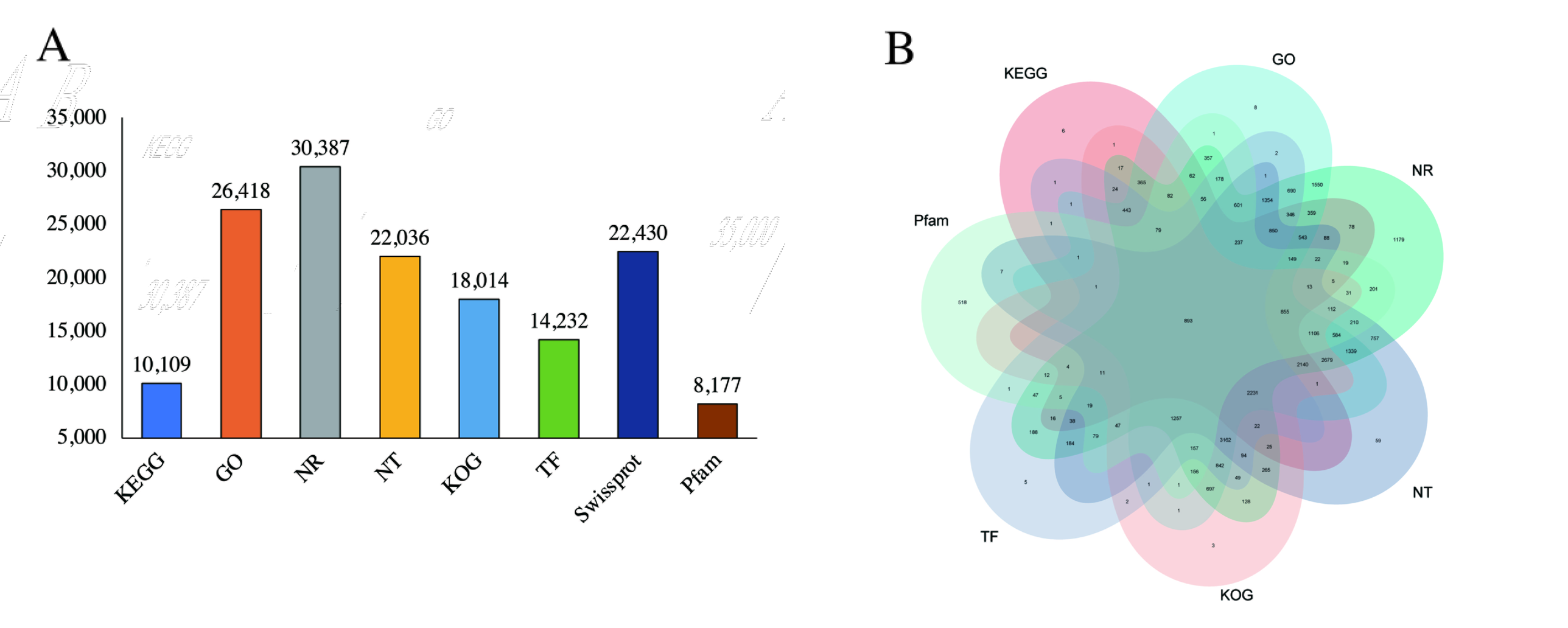

Supplement: Supplementary Figure 1 — Annotation information of the transcriptome data. [file Image1.jpeg]

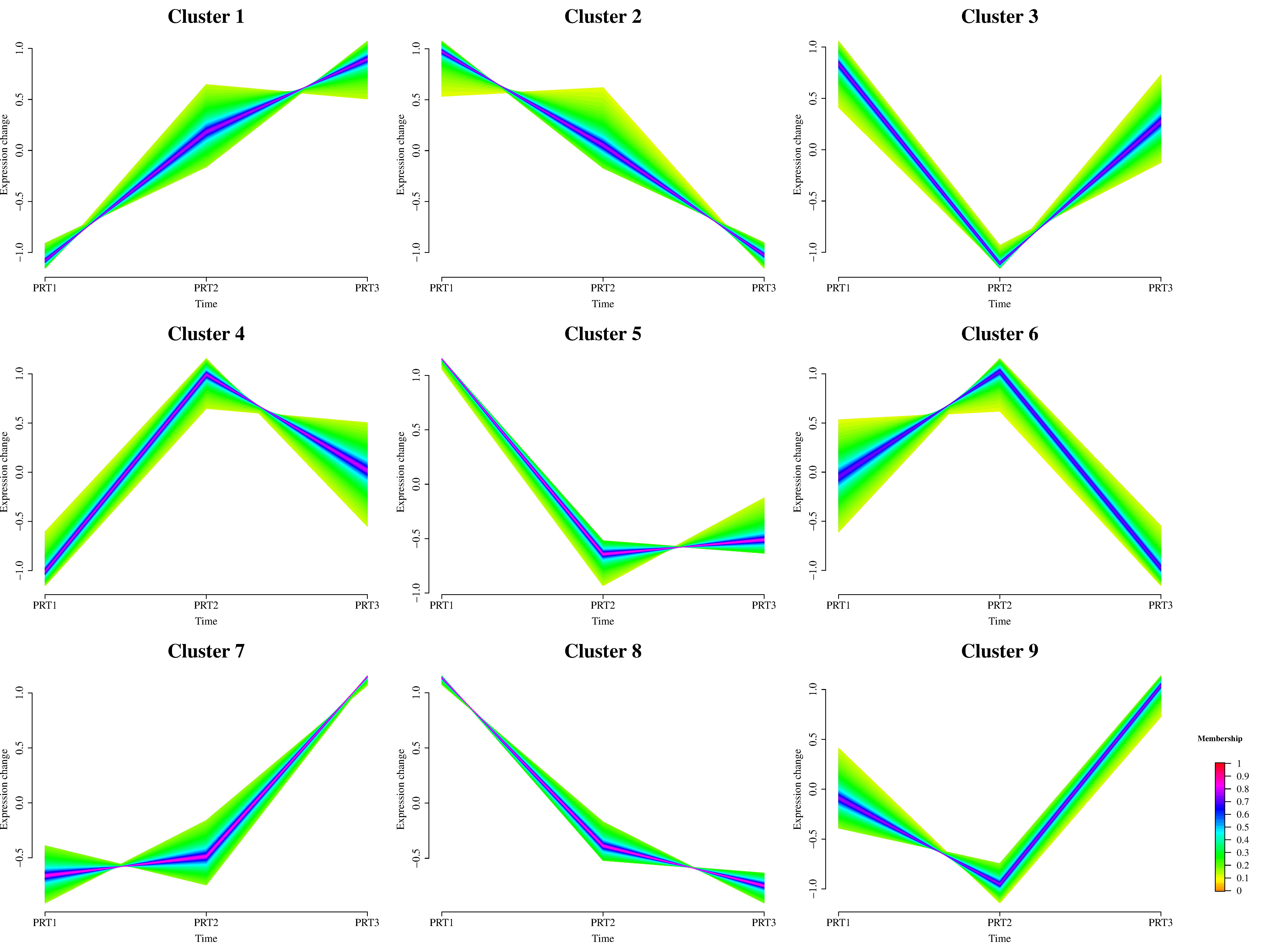

Supplement: Supplementary Figure 2 — Time-series clustering analysis results of DEGs. [file Image2.jpeg]

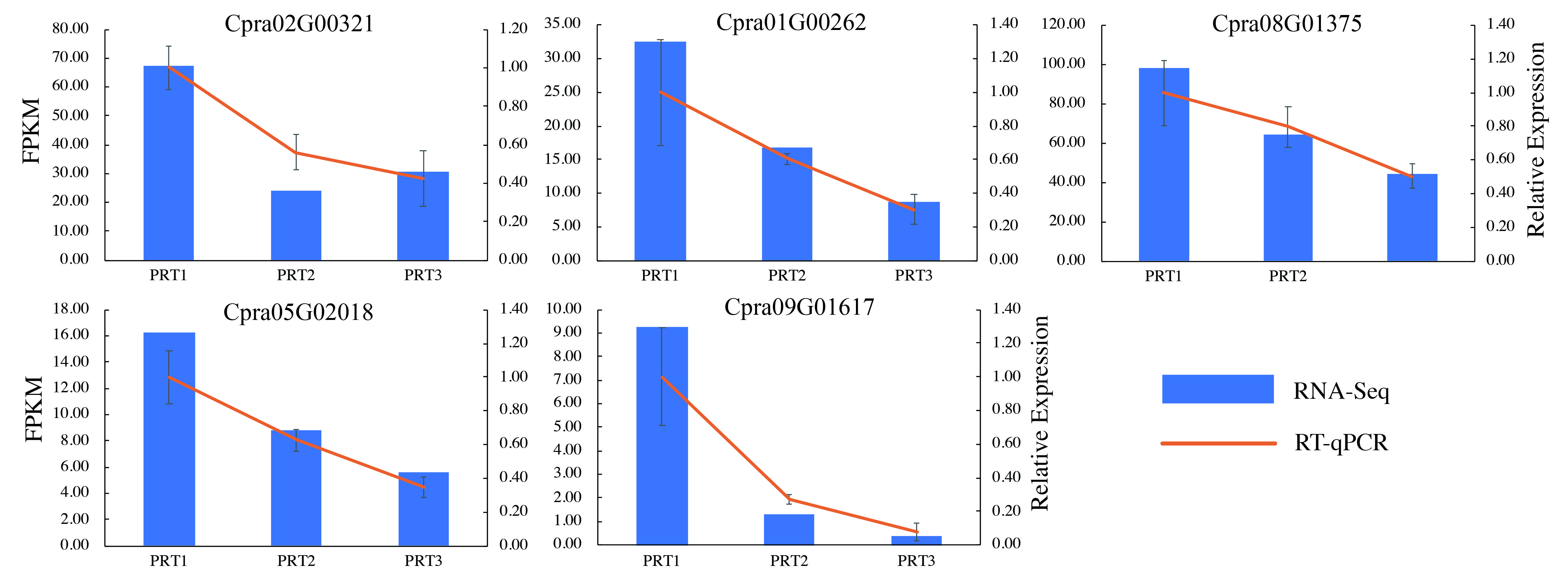

Supplement: Supplementary Figure 3 — Comparison of transcriptome sequencing results with real-time quantitative PCR results. [file Image3.jpeg]
